# Allergen reduction by nightly temperature controlled laminar airflow (TLA) decreases exacerbation rate in severe allergic asthma

**DOI:** 10.1186/2045-7022-5-S2-O5

**Published:** 2015-03-23

**Authors:** Uwe Schauer, Eckard Hamelmann

**Affiliations:** 1 Ruhr Universität Bochum, Universitäts-Kinderklinik, Bochum, Germany; 2 Ruhr Universität Bochum, Allergie Centrum, Bochum, Germany

## Introduction

It has earlier been shown that TLA significantly reduces allergen exposure and airway inflammation and improves quality of life in poorly controlled allergic asthma patients.

## Aim and objective

The primary aim was to evaluate real-life effects of TLA (Airsonett AB) when used during night-time for 12 consecutive months as add on to the patients' regular medication.

## Methods

The study compared pre- versus post-TLA initiation data from patients with poorly controlled severe persistent allergic asthma. Outcome parameters were collected at four and 12 months on medication use, asthma control, asthma symptoms, lung function, rate and use of hospital resources due to exacerbations were collected.

## Results

Data from 30 patients (mean age 28; range 8-70) completing 4 months and 27 patients completing 12 months of TLA use, are presented. Ratios (%) or means (range) At baseline After 12 months of TLA P-value Exacerbations last 12 months3.6 (0-12)1.3 (0-5)0.05Asthma Control Test14.1 (5-27)18.5 (8-27)<0.001Ratio with uncontrolled disease, GINA55.20<0.001Ratio with controlled disease, GINA10.333.3

## Conclusion

The addition of TLA to the patients' regular medication significantly reduced the risk of exacerbations and the utilization of hospital resources at disease deteriorations. Higher proportions of patients with increased control of asthma symptoms were also observed. The data demonstrate that TLA may be an important new approach in the treatment of poorly controlled allergic asthma patients.

